# Association of changes in waist circumference, waist-to-height ratio and weight-adjusted-waist index with multimorbidity among older Chinese adults: results from the Chinese longitudinal healthy longevity survey (CLHLS)

**DOI:** 10.1186/s12889-024-17846-x

**Published:** 2024-01-29

**Authors:** Zi-Ting Chen, Xiao-Meng Wang, Yi-Shi Zhong, Wen-Fang Zhong, Wei-Qi Song, Xian-Bo Wu

**Affiliations:** Department of Epidemiology, School of Public Health, Southern Medical University, Guangzhou, Guangdong 510515 China

**Keywords:** Waist circumference, Waist-to-height ratio, Weight-adjusted-waist index, Multimorbidity, Older Chinese adult

## Abstract

**Background:**

The association of changes in waist circumference (WC), waist-to-height ratio (WHtR) and weight-adjusted-waist index (WWI) with subsequent risk of multimorbidity remains unclear among older Chinese adults. Therefore, we aimed to assess this association by utilizing data from the Chinese Longitudinal Healthy Longevity Survey (CLHLS).

**Methods:**

Our study was based on the 2011/2012 wave of the CLHLS whose follow-up surveys were conducted in 2014 and 2017/2018. A total of 2900 participants aged 65 and above at baseline were enrolled. WC, WHtR, and WWI were calculated from measured height, weight, and waist circumference. Multimorbidity refers to the coexistence of two or more of 18 chronic diseases. Cox proportional hazards models were used to estimate hazard ratios (*HR*s) and 95% confidence intervals (95%*CI*s) to evaluate the effect of three-year changes in WC, WHtR, and WWI on the risk of multimorbidity.

**Results:**

During a mean follow-up time of 4.2 (2.0) years, 906 multimorbidity cases were identified. Compared to participants in the persistently low WC group, those in the WC gain group and the persistently high WC group had a higher multimorbidity risk with adjusted *HR*s (95%*CI*) of 1.23 (1.01–1.50) and 1.34(1.14–1.58), respectively. Participants in the WHtR gain group and the persistently high WHtR group also had higher risks of multimorbidity with *HR*s (95%*CI*) of 1.35 (1.08–1.67) and 1.27 (1.05–1.53), respectively, relative to the persistently low WHtR group. Compared to the persistently low WWI group, those in the WWI loss group had a lower risk of multimorbidity with *HR*s (95%*CI*) of 0.80 (0.66–0.98). For every standard deviation increase in WC, WHtR, and WWI over three years, the risk of multimorbidity was higher by 12% (95%*CI*: 1.05–1.19), 13% (95%*CI*: 1.06–1.20), and 12% (95%*CI*: 1.05–1.20), respectively.

**Conclusions:**

Associations of changes in WC, WHtR and WWI with multimorbidity are significant among older Chinese adults. The findings highlight the importance of evaluating changes in WC, WHtR, and WWI in screening and prevention of multimorbidity in older adults.

**Supplementary Information:**

The online version contains supplementary material available at 10.1186/s12889-024-17846-x.

## Introduction

The ageing process has accelerated in recent decades, and it is expected that the number of people aged 65 and above in China will increase from 172 million to 366 million from 2020 to 2050, accounting for 26.0% of the total population [1]. Aging is associated with increased susceptibility to many diseases, which can clinically lead to multimorbidity. Multimorbidity is defined as two or more chronic diseases within the same individual, which is observed in older adults ranges from 55–98% [2]. Investigations indicated that multimorbidity is rapidly becoming a norm in modern society, which can be accompanied by rising risk of premature death, functional decline, weakness, hospitalization, polypharmacy, poor quality of life and high medical costs, translating into a significant economic burden on health systems [2–5]. As the largest developing country, China also faces a huge health and economic burden of chronic diseases [6]. Therefore, early identification of high-risk population of multimorbidity, so as to prevent multimorbidity and improve prognoses has become a crucial challenge in the field of public health.

Obesity is a major health problem worldwide and has increased at an alarming rate in recent decades [7]. In China, the rate of overweight and obesity among adult residents has exceeded 50% [8]. As a known risk factor for many chronic diseases such as hypertension, diabetes, hyperlipidemia, coronary artery disease, stroke and cancer [9–11], obesity is recognized to significantly increase the risk of multimorbidity in older adults [12]. Consequently, monitoring obesity and its changes is of great medical significance for preventing the development of multimorbidity. Currently, anthropometric indices are a widely used, quick and easy public health detection tool. It is of great clinical and public health significance to find more effective anthropometric indices associated with the risk of developing multimorbidity.

Body mass index (BMI) and waist circumference (WC) are the most commonly used indicators to measure obesity. However, BMI has the disadvantage of not distinguishing between fat mass and muscle mass, so that muscular individuals are often misjudged [13, 14]. Similarly, WC is not a good indicator of obesity independent of BMI due to the high correlation with BMI [15]. Therefore, it is not reliable to evaluate obesity only by BMI or WC. Recently, waist-to-height ratio (WHtR) has been proposed as an alternative anthropometric indicator for assessing central obesity due to the effect of height on risk [16]. Previous studies have shown that WHtR has a higher correlation with abdominal fat than WC [17]. Moreover, for certain diseases, such as cardiovascular disease [18] and all-cause death [19], WHtR is a better predictor than BMI. Consequently, WHtR may be better indicator of weight management needs. In 2018, Park et al. proposed a new simple anthropometric indicator, the weight-adjusted-waist index (WWI), as a useful alternative marker of obesity and obesity-related adverse health outcomes [20]. Unlike traditional indicators of obesity, BMI or WC, WWI can reflect both fat and muscle mass components, which is a comprehensive and excellent indicator of obesity even within different BMI categories, and is best at predicting the risk of cardiac metabolic disease and mortality [20, 21].

Several studies, mainly cross-sectional studies have explored the association between obesity and multimorbidity in older adults based on anthropometric indicators, and generally indicated that obesity is a risk factor for multimorbidity [12, 22–24]. Obesity is dynamically changing and may be more relevant with multimorbidity, but studies concerning the association between changes in obesity and multimorbidity are limited, particularly among older Chinese adults. The few studies that have examined changes in obesity and multimorbidity have also shown inconsistent results. A study from China indicated that increasing WC was associated with a high risk of multimorbidity, regardless of general obesity status at baseline [25] while another study from Italy suggested that those who lost weight had a significantly increased risk of multimorbidity than other participants. In other words, the relationship between changes in obesity and multimorbidity remains controversial. In the present study, we conducted a national prospective cohort study in China to determine whether three-year changes in three key obesity indicators (WC, WHtR, and WWI) are associated with the risk of multimorbidity, thereby providing relevant evidence for early prevention of the development of multimorbidity.

## Methods

### Study design and population

The Chinese Longitudinal Healthy Longevity Survey (CLHLS) is a nationally representative prospective cohort study of older Chinese adults to investigate factors associated with human health and longevity. The baseline survey and the follow-up surveys were conducted in a randomly selected in 23 provinces of China, which accounted for about 85% of the total Chinese population. The CLHLS started in 1998 and then conducted follow-up surveys and recruited new participants to maintain the sample size in 2000, 2002, 2005, 2008/2009, 2011/2012, 2014, and 2017/2018, respectively. All centenarians were invited to be interviewed, for each centenarian interviewee, we interviewed one nearby octogenarian and one nearby nonagenarian of predefined age and sex. Whereas the surveys in 1998 and 2000 included only the oldest old, the surveys in 2002 or later, each centenarian was randomly matched with approximately 1.5 nearby younger elderly aged 65–79. More details of the study design and sampling procedures are available in the literature [26]. The study was conducted by trained professionals in participants’ homes using structured questionnaires that included demographic characteristic, socioeconomic status, lifestyle, self-assessment of health and quality of life, cognitive function, activities of daily living and so on [27, 28]. The CLHLS study was approved by the Research Ethics Committee of Peking University (IRB00001052-13074), and all participants or their surrogate respondents provided a written informed consent.

We included the sixth wave of the CLHLS, with the 2011/2012 survey as the baseline, the 2014 and 2017/2018 follow-up as the first follow-up and second follow-up, respectively. The total sample consisted of 9765 participants. Individuals aged < 65 years old (*n* = 86), with missing data on WC, WHtR, and WWI at baseline or first follow-up (*n* = 4745), with multimorbidity at baseline (*n* = 1433), without complete information on covariates (*n* = 520), and without follow-up time or outcome (*n* = 81) were excluded. Finally, 2900 participants remained for the primary analysis of the association between WC, WHtR and WWI change patterns and multimorbidity. Supplementary Fig. 1 showed the flowchart of enrollment of participants in this study.

### Exposure assessment

During the investigation, height (cm) and weight (kg) were measured by measuring tape and weighing scale without shoes and heavy clothes. When measuring WC, participants were asked to be in an upright position with a calm exhalation, and a soft tape was used to measure around the midpoint of the line between the lower rib margin and the highest point of the iliac crest. According to previous studies [29, 30], low WC was defined as WC < 85 cm in men and < 80 cm in women, while high WC was defined as ≥ 85 cm in men and ≥ 80 cm in women. We calculated WHtR using the formula: WC (cm) divided by height (cm). With reference to previous literature, WHtR was categorized into low WHtR (< 0.5) and high WHtR (≥ 0.5). WWI was calculated as WC (cm) divided by the square root of weight (kg) [20], and low WWI was defined as WWI < 11.25 cm/√kg while high WWI was defined as WWI ≥ 11.25 cm/√kg [31].

Additionally, four patterns of changes in WC, WHtR and WWI were defined using status of WC, WHtR and WWI from baseline to the first follow-up. Taking WC as an example, changes in WC can be divided into the following four groups: persistently low (low WC both at baseline and the first follow-up); WC gain (low WC at baseline but high WC at the first follow-up); WC loss (high WC at baseline but low WC at the first follow-up); persistently high (high WC both at baseline and the first follow-up). The changes in WHtR and WWI were extrapolated in the same way.

### Outcome definition

Multimorbidity refers to the coexistence of two or more chronic diseases [2]. A total of 18 chronic diseases were used to measure multimorbidity, including “hypertension”; “diabetes”; “heart disease”; “stroke”; “bronchitis”; “tuberculosis”; “cataract”; “glaucoma”; “cancer”; “gastric ulcer”; “Parkinson’s disease”; “arthritis”; “dementia”; “epilepsy”; “cholecystitis”; “blood disease”; “chronic nephritis” and “hepatitis”. Combining the above 18 diseases to assess the number of chronic diseases in participants, and those with two or more chronic diseases would be identified as multimorbidity [32, 33].

### Covariates

The covariates were selected from standardized and structured questionnaire at baseline, which included demographics, socioeconomic status, and lifestyle behaviors [34]. Demographics included age, sex, and marital status. Socioeconomic status characteristics were measured by education level, residence area, living pattern, occupation, and household income. Lifestyles covered smoking status, alcohol drinking status, physical exercise, and sleep duration. Marital status was dichotomized into married and others, those who were married with a surviving spouse were defined as married, and those who were separated, divorced, widowed, or never married were classified as others. Living pattern included two groups, namely living with household members as well as living alone or in an institution groups. Occupation referred to the main occupation before the age of 60, and was divided into farmer and non-farmer, in which farmer included farmer, farmer agriculture, forestry, animal husbandry, or fishery worker, and non-farmer included professional and technical personnel, governmental, institutional or managerial personnel, and so on. The participants were defined as smokers/drinkers if currently smoking/drinking regardless of frequency and quantity. Based on the sleep duration recommended by the National Sleep Foundation [35], participants were categorized into < 7 h, 7–9 h, and > 9 h nighttime sleep duration groups.

### Statistical analysis

At baseline, descriptive statistics were summarized by WC, WHtR, and WWI change patterns stratification. Continuous variables were reported as the mean and standard deviation (SD) whereas categorical variables as percentages. Cox proportional hazards model was used to calculate the hazard ratios (*HR*s) and 95% confidence intervals (95% *CI*s) to evaluate the association of changes in WC, WHtR and WWI with multimorbidity incidence. We developed three models to adjust for potential confounders. Model 1 was unadjusted; Model 2 was adjusted for age, sex, marital status, education level, residence, living pattern, occupation, household income; Model 3 further adjusted for smoke, drink, exercise, sleep duration in addition to Model 2. The dose-response association of changes in WC, WHtR and WWI with multimorbidity were evaluated using restricted-cubic-spline regression analysis with 4 knots. For subsequent analyses, we used similar Cox models to examine the association of changes in WC, WHtR and WWI with multimorbidity on different stages (from 0 to 1 disease to multimorbidity).

Several sensitivity analyses were performed to test the stability of the results by excluding participants who developed disability in activities of daily living (ADL) at baseline, or excluding participants with hypertension, or excluding participants with less than 2 years of follow-up. Moreover, we conducted several stratified analyses (across baseline age, sex, marital status, education level, residence, living pattern, occupation, household income, smoke, drink, exercise, and sleep duration) to examine the stratified association of changes in WC, WHtR and WWI with multimorbidity. All analyses were performed in SPSS version 27 and R4.3.1 for Windows. *P* values of < 0.05 on both sides were considered statistically significant.

## Results

### Baseline demographics characteristics

A total of 2900 participants were included in the analysis. Baseline demographics characteristics of all participants by changes in WC, WHtR and WWI are shown in Table 1. Overall, the mean (SD) age was 81.9 (10.0) years, and 49.0% were males. The mean (SD) of baseline WC, WHtR and WWI were 81.6 (11.0) cm, 0.5 (0.1), and 11.4 (1.4) cm/√kg, respectively.

**Table 1:** Baseline characteristics of all participants according to changes in WC, WHtR and WWI.

Baseline demographics characteristics	Total (*n* = 2900)	WC change, cm						WHtR change					WWI change, cm/√kg				
		Persistently low (*n* = 1091)	Gain (*n* = 425)	Loss (*n* = 531)	Persistently high (*n* = 853)	*P*	Persistently low (*n* = 622)		Gain (*n* = 487)	Loss (*n* = 554)	Persistently high (*n* = 1237)	*P*	Persistently low (*n* = 840)	Gain (*n* = 509)	Loss (*n* = 605)	Persistently high (*n* = 946)	*P*
Age	81.9 ± 10.0	83.0 ± 10.0	82.4 ± 10.4	82.9 ± 10.0	79.7 ± 9.5	< 0.001	82.4 ± 9.8		81.9 ± 10.5	82.8 ± 10.3	81.3 ± 9.8	0.022	80.2 ± 9.7	81.7 ± 10.0	81.9 ± 10.2	83.6 ± 10.0	< 0.001
Age group, years						< 0.001						0.043					< 0.001
< 80	1325 (45.7)	435 (39.9)	200 (47.1)	214 (40.3)	476 (55.8)		266 (42.8)		236 (48.5)	234 (42.2)	589 (47.6)		448 (53.3)	242 (47.5)	269 (44.5)	366 (38.7)	
≥ 80	1575 (54.3)	656 (60.1)	225 (52.9)	317 (59.7)	377 (44.2)		356 (57.2)		251 (51.5)	320 (57.8)	648 (52.4)		392 (46.7)	267 (52.5)	336 (55.5)	580 (61.3)	
Sex						< 0.001						< 0.001					< 0.001
Male	1421 (49.0)	634 (58.1)	213 (50.1)	229 (43.1)	345 (40.4)		392 (63.0)		270 (55.4)	265 (47.8)	494 (39.9)		579 (68.9)	279 (54.8)	272 (45.0)	291 (30.8)	
Female	1479 (51.0)	457 (41.9)	212 (49.9)	302 (56.9)	508 (59.6)		230 (37.0)		217 (44.6)	289 (52.2)	743 (60.1)		261 (31.1)	230 (45.2)	333 (55.0)	655 (69.2)	
Marital status						0.044						0.043					< 0.001
Married	1323 (45.6)	490 (44.9)	197 (46.4)	219 (41.2)	417 (48.9)		294 (47.3)		244 (50.1)	232 (41.9)	553 (44.7)		485 (57.7)	244 (47.9)	246 (40.7)	348 (36.8)	
Others	1577 (54.4)	601 (55.1)	228 (53.6)	312 (58.8)	436 (51.1)		328 (52.7)		243 (49.9)	322 (58.1)	684 (55.3)		355 (42.3)	265 (52.1)	359 (59.3)	598 (63.2)	
Education level, years						0.008						0.083					< 0.001
0	1554 (53.6)	575 (52.7)	241 (56.7)	304 (57.3)	434 (50.9)		308 (49.5)		253 (52.0)	318 (57.4)	675 (54.6)		362 (43.1)	266 (52.3)	330 (54.5)	596 (63.0)	
1–6	1011 (34.9)	402 (36.8)	141 (33.2)	176 (33.1)	292 (34.2)		241 (38.7)		180 (36.9)	181 (32.7)	409 (33.1)		357 (42.5)	180 (35.4)	201 (33.3)	273 (28.9)	
≥ 7	335 (11.6)	114 (10.4)	43 (10.1)	51 (9.6)	127 (14.9)		73 (11.7)		54 (11.1)	55 (9.9)	153 (12.4)		121 (14.4)	63 (12.4)	74 (12.2)	77 (8.1)	
Residence						< 0.001						< 0.001					0.003
City or town	1273 (43.9)	398 (36.5)	187 (44.0)	265 (49.9)	423 (49.6)		224 (36.0)		217 (44.6)	267 (48.2)	565 (45.7)		339 (40.4)	230 (45.2)	301 (49.8)	403 (42.6)	
Rural	1627 (56.1)	693 (63.5)	238 (56.0)	266 (50.1)	430 (50.4)		398 (64.0)		270 (55.4)	287 (51.8)	672 (54.3)		501 (59.6)	279 (54.8)	304 (50.2)	543 (57.4)	
Living pattern						0.321						0.318					0.022
With household members	2325 (80.2)	892 (81.8)	336 (79.1)	427 (80.4)	670 (78.5)		510 (82.0)		396 (81.3)	446 (80.5)	973 (78.7)		702 (83.6)	408 (80.2)	478 (79.0)	737 (77.9)	
Alone or in an institution	575 (19.8)	199 (18.2)	89 (20.9)	104 (19.6)	183 (21.5)		112 (18.0)		91 (18.7)	108 (19.5)	264 (21.3)		138 (16.4)	101 (19.8)	127 (21.0)	209 (22.1)	
Occupation						< 0.001						0.028					0.001
Farmer	2154 (74.3)	856 (78.5)	308 (72.5)	414 (78.0)	576 (67.5)		477 (76.7)		346 (71.0)	430 (77.6)	901 (72.8)		601 (71.5)	355 (69.7)	470 (77.7)	728 (77.0)	
Non-farmer	746 (25.7)	235 (21.5)	117 (27.5)	117 (22.0)	277 (32.5)		145 (23.3)		141 (29.0)	124 (22.4)	336 (27.2)		239 (28.5)	154 (30.3)	135 (22.3)	218 (23.0)	
Household income						0.014						0.090					0.067
< 10,000	997 (34.4)	405 (37.1)	157 (36.9)	172 (32.4)	263 (30.8)		235 (37.8)		176 (36.1)	175 (31.6)	411 (33.2)		296 (35.2)	187 (36.7)	181 (29.9)	333 (35.2)	
≥ 10,000	1903 (65.6)	686 (62.9)	268 (63.1)	359 (67.6)	590 (69.2)		387 (62.2)		311 (63.9)	379 (68.4)	826 (66.8)		544 (64.8)	322 (63.3)	424 (70.1)	613 (64.8)	
Smoking						< 0.001						< 0.001					< 0.001
Yes	657 (22.7)	313 (28.7)	98 (23.1)	103 (19.4)	143 (16.8)		200 (32.2)		129 (26.5)	117 (21.1)	211 (17.1)		267 (31.8)	129 (25.3)	119 (19.7)	142 (15.0)	
No	2243 (77.3)	778 (71.3)	327 (76.9)	428 (80.6)	710 (83.2)		422 (67.8)		358 (73.5)	437 (78.9)	1026 (82.9)		573 (68.2)	380 (74.7)	486 (80.3)	804 (85.0)	
Drinking						0.717						0.375					0.002
Yes	621 (21.4)	246 (22.5)	89 (20.9)	110 (20.7)	176 (20.6)		144 (23.2)		112 (23.0)	110 (19.9)	255 (20.6)		214 (25.5)	104 (20.4)	131 (21.7)	172 (18.2)	
No	2279 (78.6)	845 (77.5)	336 (79.1)	421 (79.3)	677 (79.4)		478 (76.8)		375 (77.0)	444 (80.1)	982 (79.4)		626 (74.5)	405 (79.6)	474 (78.3)	774 (81.8)	
Physical exercise						< 0.001						0.001					0.006
Yes, often	1174 (40.5)	392 (35.9)	168 (39.5)	215 (40.5)	399 (46.8)		213 (34.2)		205 (42.1)	215 (38.8)	541 (43.7)		304 (36.2)	220 (43.2)	270 (44.6)	380 (40.2)	
No, rarely	1726 (59.5)	699 (64.1)	257 (60.5)	316 (59.5)	454 (53.2)		409 (65.8)		282 (57.9)	339 (61.2)	696 (56.3)		536 (63.8)	289 (56.8)	335 (55.4)	566 (59.8)	
Sleep duration, hours						0.125						0.039					< 0.001
< 7	875 (30.2)	339 (31.1)	144 (33.9)	158 (29.8)	234 (27.4)		204 (32.8)		164 (33.7)	147 (26.5)	360 (29.1)		251 (29.9)	170 (33.4)	166 (27.4)	288 (30.4)	
7–9	1148 (39.6)	441 (40.4)	151 (35.5)	200 (37.7)	356 (41.7)		255 (41.0)		184 (37.8)	223 (40.3)	486 (39.3)		383 (45.6)	188 (36.9)	241 (39.9)	336 (35.6)	
≥ 9	877 (30.2)	311 (28.5)	130 (30.6)	173 (32.6)	263 (30.8)		163 (26.2)		139 (28.5)	184 (33.2)	391 (31.6)		206 (24.5)	151 (29.7)	198 (32.7)	322 (34.0)	
WC, cm	81.6 ± 11.0	73.1 ± 6.9	74.8 ± 6.8	88.1 ± 6.3	91.7 ± 7.9	< 0.001	71.7 ± 7.2		72.8 ± 7.4	85.4 ± 7.8	88.3 ± 8.9	< 0.001	76.5 ± 9.1	74.3 ± 9.1	87.6 ± 9.6	86.2 ± 10.0	< 0.001
WHtR	0.5 ± 0.1	0.5 ± 0.0	0.5 ± 0.0	0.6 ± 0.1	0.6 ± 0.1	< 0.001	0.5 ± 0.0		0.5 ± 0.0	0.6 ± 0.0	0.6 ± 0.1	< 0.001	0.5 ± 0.1	0.5 ± 0.1	0.6 ± 0.1	0.6 ± 0.1	< 0.001
WWI, cm/√kg	11.4 ± 1.4	10.8 ± 1.1	10.5 ± 1.1	12.4 ± 1.2	12.1 ± 1.1	< 0.001	10.4 ± 1.0		10.3 ± 1.0	12.1 ± 1.1	12.1 ± 1.1	< 0.001	10.3 ± 0.8	10.4 ± 0.8	12.2 ± 0.9	12.5 ± 1.0	< 0.001

WC, waist circumference; WHtR, waist-to-height ratio; WWI, weight-adjusted-waist index

Marital status: Married, married and living with spouse; Others, separated/divorced/widowed/never married. Occupation: Farmer, farmer agriculture, forestry, animal husbandry, or fishery worker

The distribution of all sample characteristics except household income among different WWI change groups were statistically significant (*P* < 0.05). The distribution of sample characteristics including age (years), sex, marital status, residence, occupation, smoke, and physical exercise showed statistical significance across groups with different statuses of changes in WC and WHtR. Moreover, among other characteristics, education level and household income were statistically significant among different WC change groups while sleep duration was statistically significant among different WHtR change groups (*P* < 0.05). Additionally, there was no significant difference in living pattern and drink among different WC and WHtR change groups (*P* > 0.05).

### Association of changes in WC, WHtR and WWI with multimorbidity

During a mean follow-up time of 4.2 (2.0) years, 906 participants developed multimorbidity. Table 2 presents the association between WC, WHtR and WWI change patterns and multimorbidity. In the fully adjusted model (model 3), compared to the persistently low WC group, the WC gain group had a 23% (*HR* = 1.23, 95%*CI*: 1.01–1.50) higher risk of multimorbidity and the persistently high WC group had a 34% (*HR* = 1.34, 95%*CI*: 1.14–1.58) higher risk of multimorbidity. Similarly, as compared with the persistently low WHtR group, those in the WHtR gain group had a 35% (*HR* = 1.35, 95%*CI*: 1.08–1.67) higher risk of multimorbidity and those in the persistently high WHtR group had a 27% (*HR* = 1.27, 95%*CI*: 1.05–1.53) higher risk of multimorbidity. Moreover, compared to the persistently low WWI group, those in the WWI loss group had a 20% (*HR* = 0.80, 95%*CI*: 0.66–0.98) lower risk of multimorbidity. For every standard deviation increase in WC, WHtR, and WWI over three years, the risk of multimorbidity was higher by 12% (95%*CI*: 1.05–1.19), 13% (95%*CI*: 1.06–1.20), and 12% (95%*CI*: 1.05–1.20), respectively.

**Table 2:** The association of changes in WC, WHtR, and WWI with multimorbidity

Exposure		No. of event / person years	Model 1		Model 2		Model 3	
			HR (95%CI)	*P*	HR (95%CI)	*P*	HR (95%CI)	*P*
WC change, cm	Per SD increase	906 / 12196.58	1.12 (1.05, 1.19)	0.001	1.12 (1.05, 1.19)	0.001	1.12 (1.05, 1.19)	0.001
	Persistently low (*n* = 1091)	293 / 4522.92	1 (reference)	< 0.001	1 (reference)	< 0.001	1 (reference)	< 0.001
	Gain (*n* = 425)	149 / 1777.92	1.26 (1.04, 1.54)	0.020	1.24 (1.02, 1.51)	0.035	1.23 (1.01, 1.50)	0.042
	Loss (*n* = 531)	130 / 2282.92	0.83 (0.68, 1.02)	0.082	0.82 (0.66, 1.01)	0.060	0.82 (0.67, 1.01)	0.063
	Persistently high (*n* = 853)	334 / 3612.83	1.38 (1.18, 1.61)	< 0.001	1.31 (1.11, 1.54)	0.001	1.34 (1.14, 1.58)	< 0.001
WHtR change	Per SD increase	906 / 12196.58	1.13 (1.06, 1.20)	< 0.001	1.13 (1.06, 1.20)	< 0.001	1.13 (1.06, 1.20)	< 0.001
	Persistently low (*n* = 622)	158 / 2576.00	1 (reference)	< 0.001	1 (reference)	< 0.001	1 (reference)	< 0.001
	Gain (*n* = 487)	174 / 2013.00	1.38 (1.11, 1.71)	0.004	1.34 (1.08, 1.66)	0.009	1.35 (1.08, 1.67)	0.007
	Loss (*n* = 554)	141 / 2363.25	0.89 (0.71, 1.12)	0.334	0.88 (0.70, 1.11)	0.278	0.89 (0.71, 1.12)	0.312
	Persistently high (*n* = 1237)	433 / 5244.33	1.28 (1.07, 1.54)	0.008	1.24 (1.03, 1.50)	0.022	1.27 (1.05, 1.53)	0.013
WWI change, cm/√kg	Per SD increase	906 / 12196.58	1.12 (1.05, 1.20)	0.001	1.12 (1.05, 1.20)	0.001	1.12 (1.05, 1.20)	0.001
	Persistently low (*n* = 840)	269 / 3571.67	1 (reference)	0.047	1 (reference)	0.059	1 (reference)	0.065
	Gain (*n* = 509)	176 / 2179.83	1.05 (0.87, 1.27)	0.593	1.05 (0.86, 1.27)	0.655	1.04 (0.86, 1.26)	0.685
	Loss (*n* = 605)	166 / 2624.25	0.80 (0.66, 0.97)	0.023	0.80 (0.66, 0.97)	0.026	0.80 (0.66, 0.98)	0.032
	Persistently high (*n* = 946)	295 / 3820.83	1.00 (0.85, 1.18)	0.984	0.99 (0.83, 1.18)	0.901	1.01 (0.84, 1.20)	0.951

WC, waist circumference; WHtR, waist-to-height ratio; WWI, weight-adjusted-waist index

HR, hazard ratio; CI, confidence interval; SD, standard deviation

Model 1: unadjusted

Model 2: adjusted for age, sex, marital status, education level, residence, living pattern, occupation, household income

Model 3: Model 2 + adjusted for smoke, drink, exercise, sleep duration

**Fig. 1:**
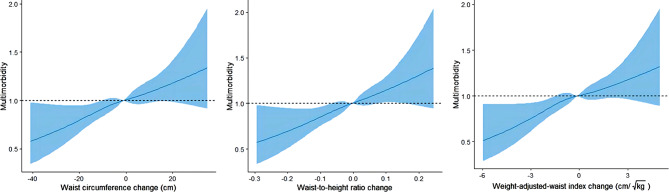
Cubic splines of three-year changes in WC, WHtR, and WWI associated with multimorbidity. Restricted-cubic-spline regression analysis with 4 knots was used to describe non-linear association of changes in waist circumference, waist-to-height ratio, and weight-adjusted-waist index with multimorbidity. Cox proportional hazards models were adjusted for age, sex, marital status, education level, residence, living pattern, occupation, household income, smoke, drink, exercise, sleep duration

We further evaluated the relationship between WC, WHtR and WWI change patterns and multimorbidity using restricted-cubic-spline analyses. The results showed no evidence of nonlinearity for the association of changes in WC (*P*_overall_=0.007, *P*_non−linearity_=0.801), WHtR (*P*_overall_=0.003, *P*_non−linearity_=0.888) and WWI (*P*_overall_=0.005, *P*_non−linearity_=0.622) and the risk of multimorbidity (Fig. 1). Specifically, the risk of multimorbidity increased with rising changes in WC, WHtR and WWI.

### From 0 to 1 disease to multimorbidity

**Fig. 2:**
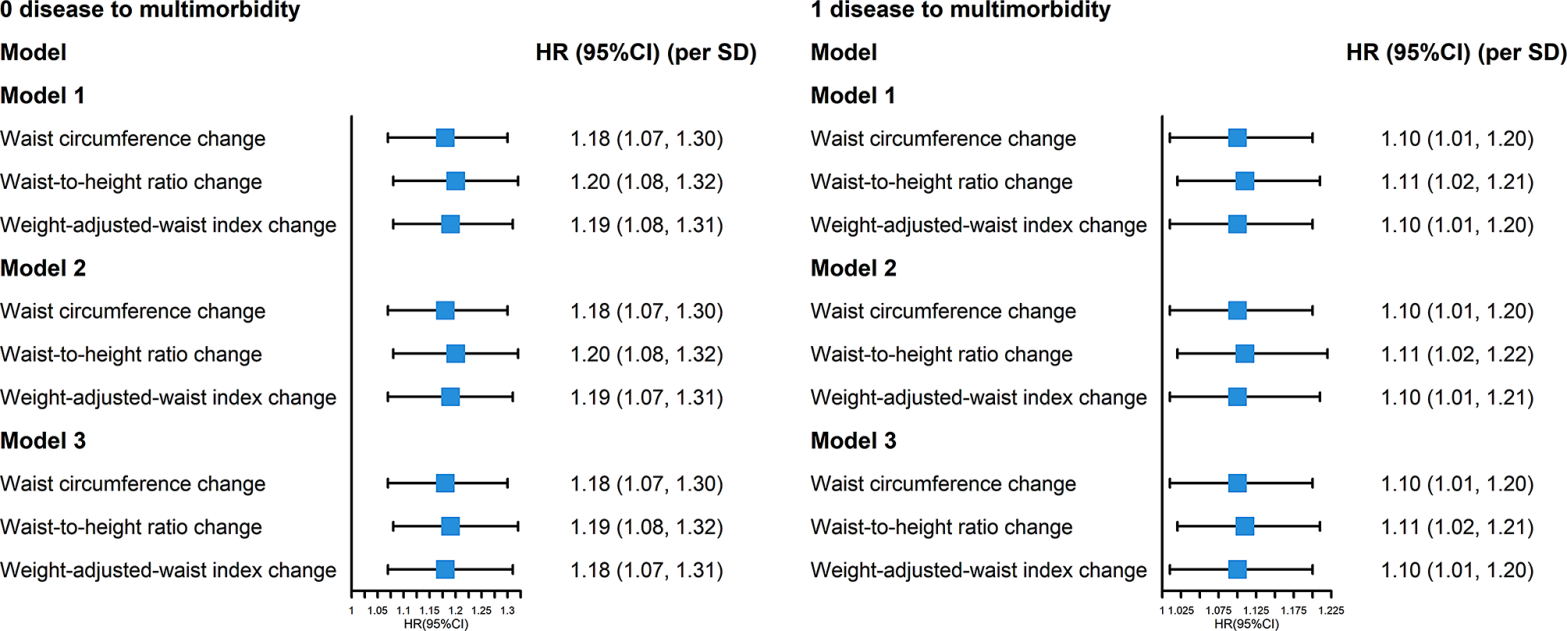
The association of changes in WC, WHtR, and WWI with multimorbidity (from 0 or 1 disease to multimorbidity, per SD).HR, hazard ratio; CI, confidence interval; SD, standard deviation. Cox proportional hazards models were adjusted for age, sex, marital status, education level, residence, living pattern, occupation, household income, smoke, drink, exercise, sleep duration

A total of 1596 participants without 18 included diseases at baseline were included in the stage of from 0 disease to multimorbidity. During a mean follow-up time of 4.3 (2.1) years, 368 participants developed multimorbidity. In the fully adjusted model (model 3), for every standard deviation increase in WC, WHtR and WWI change, the risk of multimorbidity increased by 18% (*HR* = 1.18, 95%*CI*: 1.07–1.30), 19% (*HR* = 1.19, 95%*CI*: 1.08–1.32), and 18% (*HR* = 1.18, 95%*CI*: 1.07–1.31), respectively (Fig. 2). Additionally, compared with older adults in the persistently low WC group, those in the WC gain group had a 34% (*HR* = 1.34, 95%*CI*: 1.01–1.78) higher risk of multimorbidity. Likewise, compared with older adults in the persistently low WHtR group, those in the WHtR gain group had a 39% (*HR* = 1.39, 95%*CI*: 1.00-1.92) higher risk of multimorbidity (Supplementary Fig. 2).

Among 1304 participants in the stage of from 1 disease to multimorbidity, 538 participants developed multimorbidity during a mean follow-up time of 4.1 (2.0) years. In the fully adjusted model (model 3), for every standard deviation increase in WC, WHtR, and WWI change, the risk of multimorbidity increased by 10% (*HR* = 1.10, 95%*CI*: 1.01–1.20), 11% (*HR* = 1.11, 95%*CI*: 1.02–1.21) and 10% (*HR* = 1.10, 95%*CI*: 1.01–1.20), respectively (Fig. 2), with increased risk less than the stage of from 0 disease to multimorbidity. In addition, compared with older adults in the persistently low WC group, those in the persistently high WC group had a 31% (*HR* = 1.31, 95%*CI*: 1.06–1.61) higher risk of multimorbidity. Compared with older adults in the persistently low WHtR group, those in the WHtR gain group had a 36% (*HR* = 1.36, 95%*CI*: 1.01–1.82) higher risk of multimorbidity (Supplementary Fig. 2).

### Subgroup and sensitivity analyses

We performed subgroup analysis by age, sex, marital status, educational level, residence, living pattern, occupation, household income, smoke, drink, physical exercise and sleep duration. We found that none of the covariates significantly modified the association between WC (Supplementary Fig. 3) or WHtR (Supplementary Fig. 4) and the risk of multimorbidity (*P* for interaction > 0.05). However, the association between change in WWI and multimorbidity were more pronounced in the older adults living with household members (*P* for interaction = 0.042). Compared with the persistently low WWI group, the WWI loss group had a 25% (*HR* = 0.75, 95%*CI*: 0.61–0.94) lower risk of multimorbidity. Moreover, the interaction between change in WWI and drinking status was significant for multimorbidity outcomes (*P* for interaction = 0.026). Compared with the persistently low WWI group, the WWI gain group had a 58% (*HR* = 1.58, 95%*CI*: 1.02–2.45) higher risk of multimorbidity within current drinkers, while the WWI loss group had a 27% (*HR* = 0.73, 95%*CI*: 0.59–0.91) lower risk of multimorbidity among non-drinkers (Supplementary Fig. 5).

Sensitivity analyses revealed no substantial change after excluding participants with ADL disability (Supplementary Table 1; Supplementary Fig. 6) or participants with hypertension (Supplementary Table 2; Supplementary Fig. 7) or participants with less than 2 years of follow-up (Supplementary Table 3; Supplementary Fig. 8).

## Discussion

The present study focused on the association of changes in WC, WHtR, and WWI with multimorbidity among older Chinese adults. Our results showed that rising changes in WC, WHtR, and WWI were associated with an increased risk of multimorbidity. Compared with participants in the persistently low group of WC, WHtR, those in the gain group and the persistently high group of WC, WHtR had significantly higher multimorbidity risk. Moreover, compared with the persistently low WWI group, the WWI loss group was correlated with a lower risk of multimorbidity. The findings advance our understandings of the complex association between anthropometric changes and multimorbidity, and highlight the importance of strengthening the health management of older adults and maintain optimal body shape to prevent adverse health outcomes.

The association between adiposity and multimorbidity has been extensively explored in epidemiological studies [12, 23, 24, 36–38]. For instance, a systematic review and meta-analysis of 8 longitudinal studies found a higher risk of multimorbidity among individuals with obesity [36]. Similarly, a cross-sectional study involving 20,198 participants from China, India, Ghana, Mexico, Russia and South Africa reported that significant positive association between obesity (measured by BMI and WC) and 5 out of 12 included chronic conditions [24]. Although studies have consistently demonstrated that obesity is positively associated with multimorbidity, the studies on the relationship between obesity indicators change and multimorbidity are limited and still controversial. In our study, we found both WC and WHtR gain were associated with higher risk of multimorbidity. Consistent with our findings, a cohort study showed that when participants with normal baseline WC progressed to central obesity during follow-up, the risk of multimorbidity was significantly increased (*HR* = 1.78, 95% *CI*: 1.64–1.95) [25]. However, another study reported that obese participants who lost weight over follow-up had a significantly greater increase in multimorbidity than other participants, perhaps related to reverse causality, where certain chronic conditions can lead to weight loss in patients during the preclinical stage [39, 40].

In this study, we first explored the association between WHtR, WWI change patterns and multimorbidity. WC and WHtR are considered to be important anthropometric indicators of abdominal obesity [41, 42]. Previous studies have suggested that WC and WHtR can reflect body fat percentage accurately and play an important role in predicting some chronic diseases, such as cardiovascular disease and metabolic syndrome [43]. The pathway may explain that abdominal obesity significantly increased plasma triglycerides, low density lipoproteins and very low density lipoproteins of body, which have been shown to increase the risk of adverse outcomes such as cardiovascular disease, diabetes, hypertension, and kidney diseases [44]. In addition, people with abdominal obesity tend to have excess visceral fat, which can lead to high doses of adipokines from the portal vein to the liver and other body tissues, causing a variety of chronic diseases [45]. As the newly proposed simple indicator of obesity, WWI could reflect both fat and muscle mass components [21]. It is intuitive to suggest that increased fat mass and decreased skeletal muscle mass may be associated with more inflammation, which can lead to increased chronic diseases [46, 47]. Previous studies have highlighted that visceral adipose tissue produces large amounts of interleukin-6 (IL-6), which promotes the secretion of acute-phase proteins such as C-reactive protein (CRP), and thus the levels of IL-6 and CRP are significantly increased in individuals with abdominal obesity [48]. A cross-sectional study showed that the number of chronic diseases was linearly associated with the levels of inflammatory markers IL-6 and CRP [49]. Therefore,WWI loss may reduce inflammation and thus the risk of multimorbidity, which could reasonably explain our results. Moreover, the persistently low WWI group included those who had always been underweight, and underweight older adults were prone to malnutrition, which is associated with some noninfectious chronic diseases [50]. This may explain why the risk of multimorbidity was higher in the persistently low WWI group than in the WWI loss group.

Our study showed that elevated WC, WHtR, and WWI were significantly associated with increased risk of multimorbidity both at baseline of 0 disease and 1 disease. Notably, strategies that move from baseline of 0 or 1 disease to multimorbidity assessments can provide mutual validation and increase the reliability of the association.

In addition, we conducted a subgroup analysis of covariates, and the results showed that in older adults living with family members, the loss of WWI had a more significant effect on reducing the risk of multimorbidity, which may be related to the fact that family companionship can reduce social loneliness and isolation in older adults [51]. This association was also found in non-drinkers, but in drinkers, WWI gain was more significant in increasing the risk of multimorbidity, this finding agreed with existing study showing that alcohol and obesity are risk factors for certain chronic diseases, and they often co-exist [52]. In detail, alcohol consumption is associated with an increased risk of cancer, cardiovascular disease, neurological disease, and more [53, 54].

### Strengths and limitations

The study has several strengths. First, weight, height, and waist circumference measurements were assessed by trained researchers rather than self-reported, which decreased the misclassification bias of self-reported measurements. Second, the identification of multimorbidity was comparatively accurate and comprehensive, including self-reported chronic diseases by participants and diagnoses made by doctors based on thorough professional examination. Third, it is one of the few studies to measure the association between longitudinal changes in obesity indicators and multimorbidity in a population-based sample, providing additional insights into changes in obesity and multimorbidity.

However, our study also has some limitations. First, we cannot distinguish between intentional and unintentional changes in WC, WHtR, and WWI. Second, the results depend to some extent on the definition of outcome. Our definition of multimorbidity included 18 diseases, while some chronic diseases, such as asthma and chronic obstructive pulmonary disease, are not included. In addition, disease severity was not considered as part of the definition of multimorbidity. Third, the use of self-reported profiles of various diseases rather than objective indicators of multimorbidity may lead to recall bias in data on multimorbidity. In addition, although the causal association was well validated in prospective cohort studies, chronic diseases may cause loss in WC, WHtR, and WWI, and the drugs used to treat the chronic disease may cause gain in WC, WHtR, and WWI. Finally, although we adjusted for many covariates, residual and unmeasured confounding factors cannot be completely excluded.

## Conclusions

The study found that changes in WC, WHtR, and WWI in older Chinese adults were associated with multimorbidity. The findings indicate that WC, WHtR and WWI change are important independent and modifiable risk factors for multimorbidity. Measuring WC, WHtR, and WWI and following up on their changes in health screenings may provide incremental benefits for multimorbidity screening and preventing in older adults.

### Electronic supplementary material

Below is the link to the electronic supplementary material.

Supplementary Material 1

## Data Availability

PKU Centre for Healthy Ageing and Development. 2021. “Chinese Longitudinal Healthy Longevity Survey (CLHLS).” Peking University Open Research Data. 10.18170/DVN/WBO7LK. The datasets used are available from the first author on reasonable request.

## Abbreviations

CLHLS: Chinese Longitudinal Healthy Longevity Survey
WC: waist circumference
WHtR: waist-to-height ratio
WWI: weight-adjusted-waist index
HRs: hazard ratios
CIs: confidence intervals
BMI: body mass index
SD: standard deviation
ADL: activities of daily living
IL-6: interleukin-6
CRP: C-reactive protein
