# Supplementary material for: Association of changes in waist circumference, waist-to-height ratio and weight-adjusted-waist index with multimorbidity among older Chinese adults: results from the Chinese longitudinal healthy longevity survey (CLHLS)
Source: BMC Public Health. 2024 Jan 29;24:318. doi: 10.1186/s12889-024-17846-x (PMC10825986; doi:10.1186/s12889-024-17846-x)
Supplement: Supplementary file 1 — Supplementary Material 1 [file 12889_2024_17846_MOESM1_ESM.docx]

**Association of changes in waist circumference, waist-to-height ratio and weight-adjusted-waist index with multimorbidity among Chinese older adults: Results from the Chinese Longitudinal Healthy Longevity Survey (CLHLS)**

**Supplementary Materials**

Supplementary Table 1. Sensitivity analyses of the association of changes in WC, WHtR, and WWI with multimorbidity (excluding participants with ADL disability).

Supplementary Table 2. Sensitivity analyses of the association of changes in WC, WHtR, and WWI with multimorbidity (excluding participants with hypertension).

Supplementary Table 3. Sensitivity analyses of the association of changes in WC, WHtR, and WWI with multimorbidity (excluding participants with less than 2 years of follow-up).

Supplementary Figure 1. Flowchart of participant recruitment.

Supplementary Figure 2. The association of changes in WC, WHtR, and WWI with multimorbidity (from 0 or 1 disease to multimorbidity, four change patterns).

Supplementary Figure 3. Association between changes in WC and multimorbidity in subgroups.

Supplementary Figure 4. Association between changes in WHtR and multimorbidity in subgroups.

Supplementary Figure 5. Association between changes in WWI and multimorbidity in subgroups.

Supplementary Figure 6. Cubic splines of three-year changes in WC, WHtR, and WWI associated with multimorbidity (excluding participants with ADL disability).

Supplementary Figure 7. Cubic splines of three-year changes in WC, WHtR, and WWI associated with multimorbidity (excluding participants with hypertension).

Supplementary Figure 8. Cubic splines of three-year changes in WC, WHtR, and WWI associated with multimorbidity (excluding participants with less than 2 years of follow-up).

Supplementary Table 1. Sensitivity analyses of the association of changes in WC, WHtR, and WWI with multimorbidity (excluding participants with ADL disability).

| Exposure | | No. of event / person years | Model 1 | | Model 2 | | Model 3 | |
| --- | --- | --- | --- | --- | --- | --- | --- | --- |
|  |  |  | HR (95%CI) | *P* | HR (95%CI) | *P* | HR (95%CI) | *P* |
| WC change, cm | Per SD increase | 791 / 10915.92 | 1.13 (1.05, 1.21) | 0.001 | 1.13 (1.05, 1.21) | 0.001 | 1.13 (1.05, 1.21) | 0.001 |
|  | Persistently low (n=940) | 241 / 4012.92 | 1 (reference) | < 0.001 | 1 (reference) | < 0.001 | 1 (reference) | < 0.001 |
|  | Gain (n=365) | 130 / 1568.50 | 1.35 (1.09, 1.67) | 0.007 | 1.31 (1.06, 1.63) | 0.013 | 1.30 (1.05, 1.61) | 0.016 |
|  | Loss (n=467) | 117 / 2055.67 | 0.90 (0.72, 1.12) | 0.350 | 0.88 (0.70, 1.10) | 0.264 | 0.88 (0.70, 1.10) | 0.259 |
|  | Persistently high (n=757) | 303 / 3278.83 | 1.50 (1.26, 1.77) | < 0.001 | 1.42 (1.19, 1.69) | < 0.001 | 1.45 (1.21, 1.72) | < 0.001 |
| WHtR change | Per SD increase | 791 / 10915.92 | 1.14 (1.06, 1.22) | < 0.001 | 1.14 (1.06, 1.23) | < 0.001 | 1.14 (1.07, 1.23) | < 0.001 |
|  | Persistently low (n=531) | 123 / 2272.25 | 1 (reference) | < 0.001 | 1 (reference) | < 0.001 | 1 (reference) | < 0.001 |
|  | Gain (n=420) | 152 / 1778.00 | 1.55 (1.22, 1.96) | < 0.001 | 1.50 (1.18, 1.91) | 0.001 | 1.51 (1.19, 1.92) | 0.001 |
|  | Loss (n=488) | 126 / 2133.67 | 1.01 (0.78, 1.29) | 0.970 | 0.99 (0.77, 1.27) | 0.923 | 1.00 (0.78, 1.28) | 0.975 |
|  | Persistently high (n=1090) | 390 / 4732.00 | 1.46 (1.19, 1.78) | < 0.001 | 1.41 (1.15, 1.73) | 0.001 | 1.44 (1.17, 1.77) | 0.001 |
| WWI change, cm/√kg | Per SD increase | 791 / 10915.92 | 1.13 (1.05, 1.21) | 0.001 | 1.13 (1.05, 1.22) | 0.001 | 1.13 (1.05, 1.22) | 0.001 |
|  | Persistently low (n=737) | 225 / 3218.33 | 1 (reference) | 0.091 | 1 (reference) | 0.109 | 1 (reference) | 0.106 |
|  | Gain (n=444) | 155 / 1932.83 | 1.13 (0.92, 1.39) | 0.239 | 1.13 (0.92, 1.38) | 0.261 | 1.12 (0.91, 1.38) | 0.271 |
|  | Loss (n=537) | 149 / 2360.50 | 0.86 (0.70, 1.06) | 0.161 | 0.86 (0.70, 1.07) | 0.173 | 0.87 (0.70, 1.08) | 0.199 |
|  | Persistently high (n=811) | 262 / 3404.25 | 1.07 (0.89, 1.28) | 0.471 | 1.06 (0.88, 1.28) | 0.549 | 1.08 (0.89, 1.31) | 0.417 |

WC, waist circumference; WHtR, waist-to-height ratio; WWI, weight-adjusted-waist index.

HR, hazard ratio; CI, confidence interval; SD, standard deviation.

Model 1: unadjusted.

Model 2: adjusted for age, sex, marital status, education level, residence, living pattern, occupation, household income.

Model 3: Model 2 + adjusted for smoke, drink, exercise, sleep duration.

Supplementary Table 2. Sensitivity analyses of the association of changes in WC, WHtR, and WWI with multimorbidity (excluding participants with hypertension).

| Exposure | | No. of event / person years | Model 1 | | Model 2 | | Model 3 | |
| --- | --- | --- | --- | --- | --- | --- | --- | --- |
|  |  |  | HR (95%CI) | *P* | HR (95%CI) | *P* | HR (95%CI) | *P* |
| WC change, cm | Per SD increase | 748 / 13902.08 | 1.12 (1.04, 1.20) | 0.002 | 1.12 (1.04, 1.20) | 0.002 | 1.12 (1.04, 1.20) | 0.002 |
|  | Persistently low (n=1165) | 234 / 4914.83 | 1 (reference) | < 0.001 | 1 (reference) | < 0.001 | 1 (reference) | < 0.001 |
|  | Gain (n=476) | 125 / 2030.83 | 1.24 (1.00, 1.54) | 0.051 | 1.20 (0.97, 1.50) | 0.099 | 1.19 (0.96, 1.48) | 0.116 |
|  | Loss (n=592) | 100 / 2612.92 | 0.76 (0.60, 0.96) | 0.020 | 0.73 (0.58, 0.93) | 0.009 | 0.73 (0.58, 0.93) | 0.010 |
|  | Persistently high (n=1009) | 289 / 4343.50 | 1.36 (1.14, 1.61) | < 0.001 | 1.26 (1.06, 1.51) | 0.011 | 1.29 (1.07, 1.54) | 0.006 |
| WHtR change | Per SD increase | 748 / 13902.08 | 1.12 (1.04, 1.20) | 0.003 | 1.12 (1.04, 1.20) | 0.002 | 1.12 (1.04, 1.20) | 0.003 |
|  | Persistently low (n=658) | 140 / 2774.83 | 1 (reference) | < 0.001 | 1 (reference) | 0.001 | 1 (reference) | 0.001 |
|  | Gain (n=529) | 133 / 2279.17 | 1.34 (1.05, 1.71) | 0.018 | 1.31 (1.02, 1.67) | 0.033 | 1.32 (1.03, 1.69) | 0.026 |
|  | Loss (n=608) | 114 / 2648.42 | 0.90 (0.70, 1.17) | 0.428 | 0.88 (0.68, 1.14) | 0.346 | 0.90 (0.70, 1.17) | 0.433 |
|  | Persistently high (n=1447) | 373 / 6199.67 | 1.32 (1.08, 1.62) | 0.008 | 1.27 (1.03, 1.56) | 0.026 | 1.30 (1.05, 1.60) | 0.016 |
| WWI change, cm/√kg | Per SD increase | 748 / 13902.08 | 1.12 (1.04, 1.21) | 0.002 | 1.12 (1.05, 1.21) | 0.002 | 1.12 (1.04, 1.20) | 0.002 |
|  | Persistently low (n=934) | 223 / 4084.08 | 1 (reference) | 0.186 | 1 (reference) | 0.194 | 1 (reference) | 0.236 |
|  | Gain (n=571) | 149 / 2474.42 | 1.06 (0.86, 1.30) | 0.594 | 1.05 (0.85, 1.29) | 0.659 | 1.04 (0.84, 1.28) | 0.733 |
|  | Loss (n=671) | 140 / 2945.92 | 0.83 (0.67, 1.02) | 0.081 | 0.82 (0.67, 1.02) | 0.077 | 0.83 (0.67, 1.03) | 0.086 |
|  | Persistently high (n=1066) | 236 / 4397.67 | 0.96 (0.80, 1.16) | 0.696 | 0.95 (0.79, 1.15) | 0.619 | 0.96 (0.80, 1.17) | 0.707 |

WC, waist circumference; WHtR, waist-to-height ratio; WWI, weight-adjusted-waist index.

HR, hazard ratio; CI, confidence interval; SD, standard deviation.

Model 1: unadjusted.

Model 2: adjusted for age, sex, marital status, education level, residence, living pattern, occupation, household income.

Model 3: Model 2 + adjusted for smoke, drink, exercise, sleep duration.

Supplementary Table 3. Sensitivity analyses of the association of changes in WC, WHtR, and WWI with multimorbidity (excluding participants with less than 2 years of follow-up).

| Exposure | | No. of event / person years | Model 1 | | Model 2 | | Model 3 | |
| --- | --- | --- | --- | --- | --- | --- | --- | --- |
|  |  |  | HR (95%CI) | *P* | HR (95%CI) | *P* | HR (95%CI) | *P* |
| WC change, cm | Per SD increase | 861 / 11922.50 | 1.11 (1.04, 1.19) | 0.002 | 1.11 (1.04, 1.19) | 0.002 | 1.11 (1.04, 1.19) | 0.002 |
|  | Persistently low (n=1023) | 274 / 4394.67 | 1 (reference) | < 0.001 | 1 (reference) | < 0.001 | 1 (reference) | < 0.001 |
|  | Gain (n=399) | 141 / 1728.83 | 1.28 (1.04, 1.57) | 0.018 | 1.25 (1.02, 1.53) | 0.034 | 1.23 (1.01, 1.52) | 0.044 |
|  | Loss (n=516) | 128 / 2254.42 | 0.87 (0.71, 1.08) | 0.204 | 0.85 (0.69, 1.06) | 0.145 | 0.85 (0.69, 1.06) | 0.145 |
|  | Persistently high (n=817) | 318 / 3544.58 | 1.40 (1.19, 1.64) | < 0.001 | 1.32 (1.11, 1.56) | 0.001 | 1.35 (1.14, 1.59) | < 0.001 |
| WHtR change | Per SD increase | 861 / 11922.50 | 1.12 (1.05, 1.20) | < 0.001 | 1.12 (1.05, 1.20) | 0.001 | 1.12 (1.05, 1.20) | 0.001 |
|  | Persistently low (n=582) | 146 / 2500.58 | 1 (reference) | < 0.001 | 1 (reference) | < 0.001 | 1 (reference) | < 0.001 |
|  | Gain (n=460) | 166 / 1962.25 | 1.42 (1.14, 1.78) | 0.002 | 1.37 (1.10, 1.71) | 0.006 | 1.38 (1.10, 1.72) | 0.005 |
|  | Loss (n=542) | 138 / 2340.50 | 0.94 (0.75, 1.19) | 0.616 | 0.93 (0.73, 1.17) | 0.515 | 0.93 (0.74, 1.18) | 0.558 |
|  | Persistently high (n=1171) | 411 / 5119.17 | 1.31 (1.09, 1.59) | 0.005 | 1.26 (1.04, 1.53) | 0.017 | 1.29 (1.06, 1.56) | 0.011 |
| WWI change, cm/√kg | Per SD increase | 861 / 11922.50 | 1.11 (1.04, 1.19) | 0.002 | 1.11 (1.04, 1.19) | 0.002 | 1.11 (1.04, 1.19) | 0.002 |
|  | Persistently low (n=798) | 250 / 3492.50 | 1 (reference) | 0.109 | 1 (reference) | 0.133 | 1 (reference) | 0.131 |
|  | Gain (n=473) | 165 / 2111.75 | 1.06 (0.87, 1.29) | 0.555 | 1.05 (0.86, 1.28) | 0.605 | 1.05 (0.86, 1.27) | 0.666 |
|  | Loss (n=593) | 162 / 2601.42 | 0.84 (0.69, 1.02) | 0.078 | 0.84 (0.68, 1.03) | 0.085 | 0.84 (0.69, 1.03) | 0.091 |
|  | Persistently high (n=891) | 284 / 3716.83 | 1.04 (0.88, 1.23) | 0.657 | 1.03 (0.86, 1.23) | 0.744 | 1.04 (0.87, 1.25) | 0.637 |

WC, waist circumference; WHtR, waist-to-height ratio; WWI, weight-adjusted-waist index.

HR, hazard ratio; CI, confidence interval; SD, standard deviation.

Model 1: unadjusted.

Model 2: adjusted for age, sex, marital status, education level, residence, living pattern, occupation, household income.

Model 3: Model 2 + adjusted for smoke, drink, exercise, sleep duration.


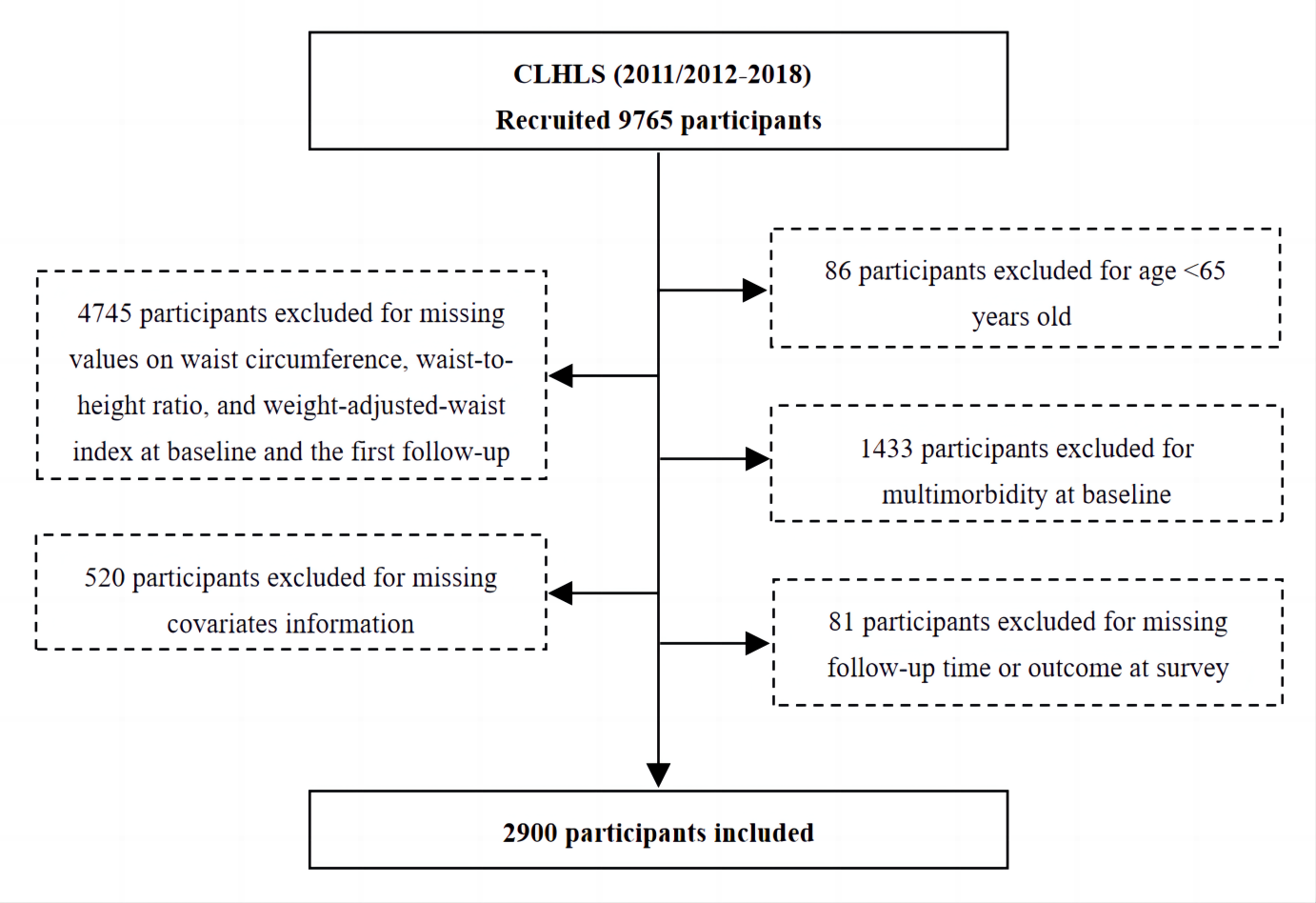


Supplementary Figure 1. Flowchart of participant recruitment.


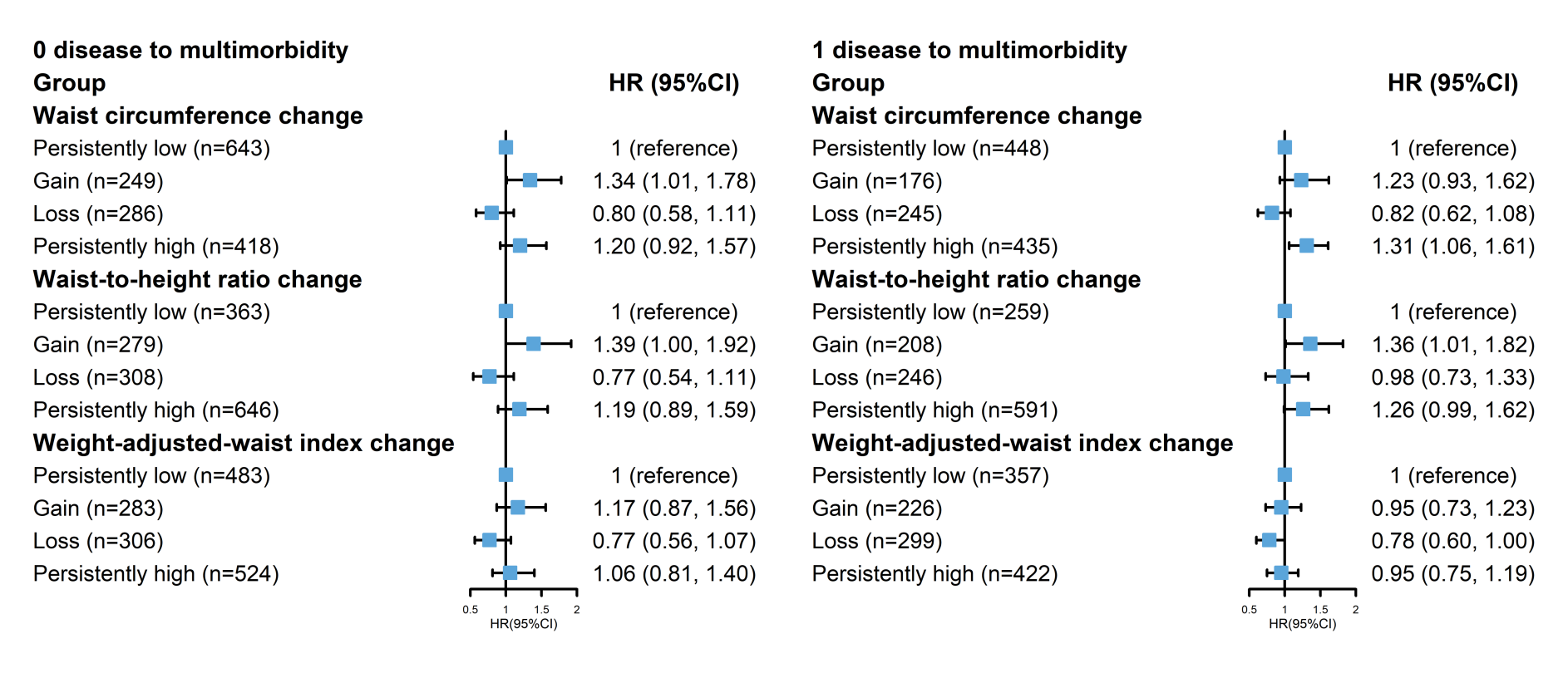


Supplementary Figure 2. The association of changes in WC, WHtR, and WWI with multimorbidity (from 0 or 1 disease to multimorbidity, four change patterns).

HR, hazard ratio; CI, confidence interval.

Cox proportional hazards models were adjusted for age, sex, marital status, education level, residence, living pattern, occupation, household income, smoke, drink, exercise, sleep duration.


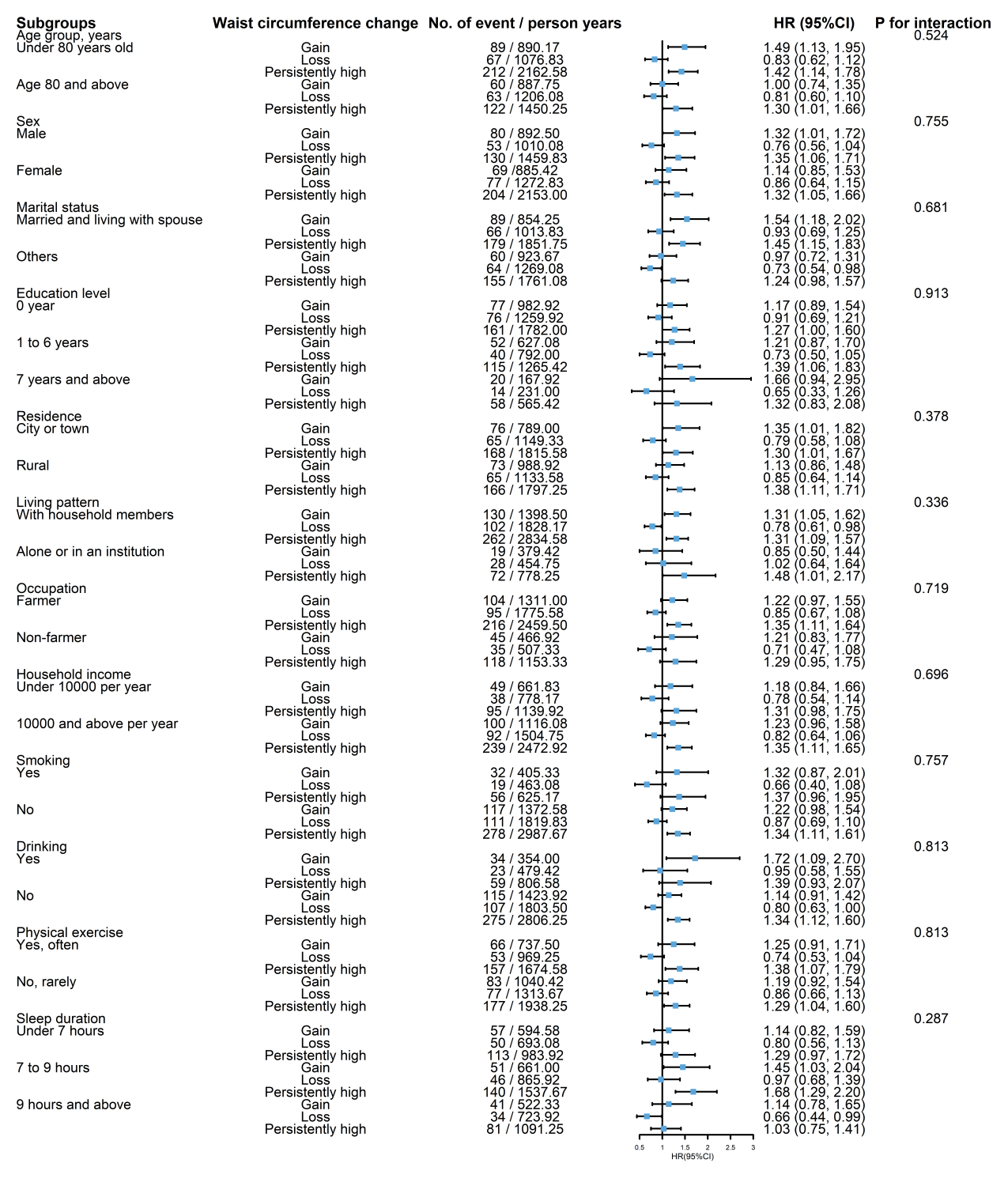
Supplementary Figure 3. Association between changes in WC and multimorbidity in subgroups.

HR, hazard ratio; CI, confidence interval.

Cox proportional hazards models were adjusted for age, sex, marital status, education level, residence, living pattern, occupation, household income, smoke, drink, exercise, sleep duration.


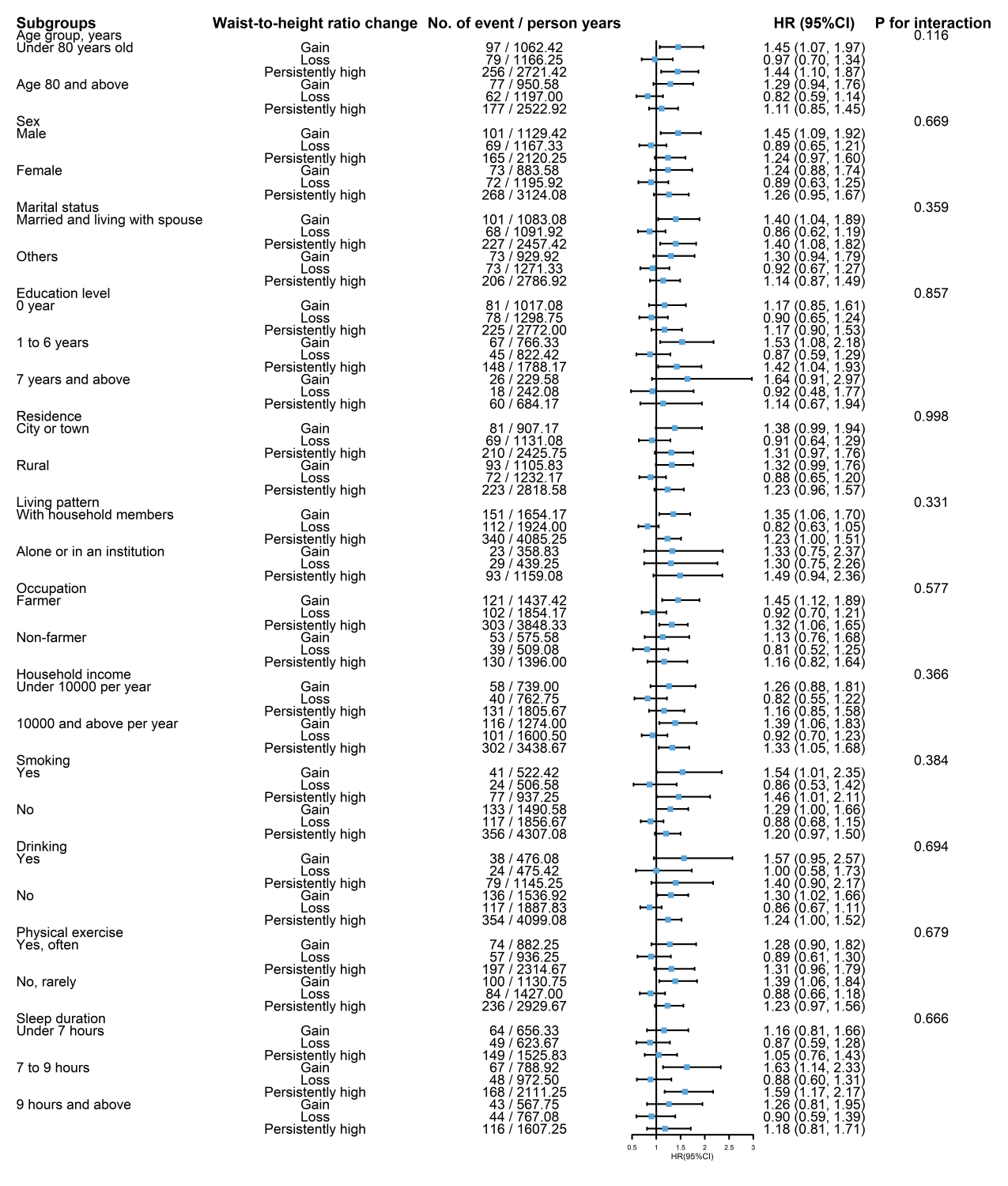


Supplementary Figure 4. Association between changes in WHtR and multimorbidity in subgroups.

HR, hazard ratio; CI, confidence interval.

Cox proportional hazards models were adjusted for age, sex, marital status, education level, residence, living pattern, occupation, household income, smoke, drink, exercise, sleep duration.


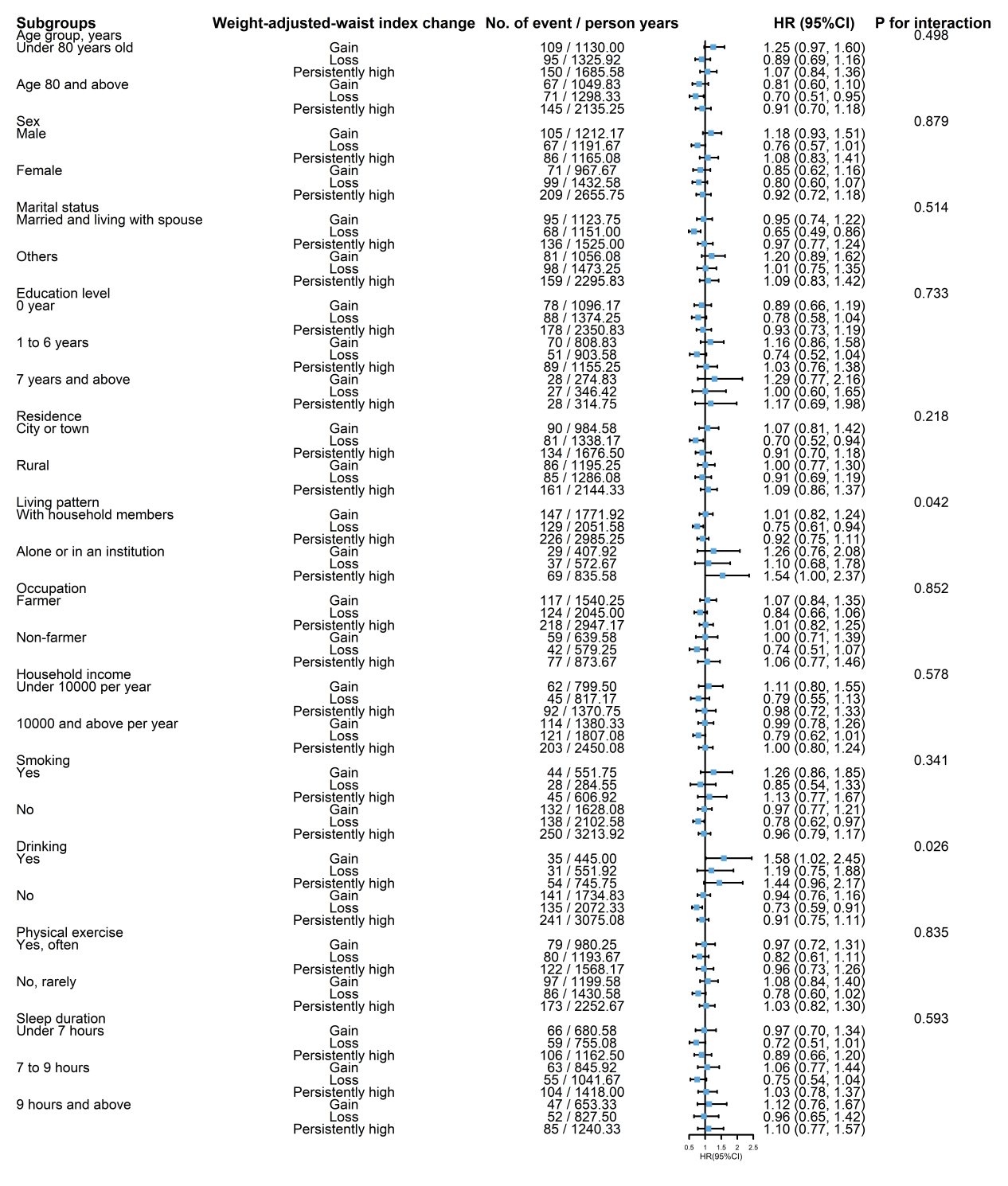


Supplementary Figure 5. Association between changes in WWI and multimorbidity in subgroups.

HR, hazard ratio; CI, confidence interval.

Cox proportional hazards models were adjusted for age, sex, marital status, education level, residence, living pattern, occupation, household income, smoke, drink, exercise, sleep duration.


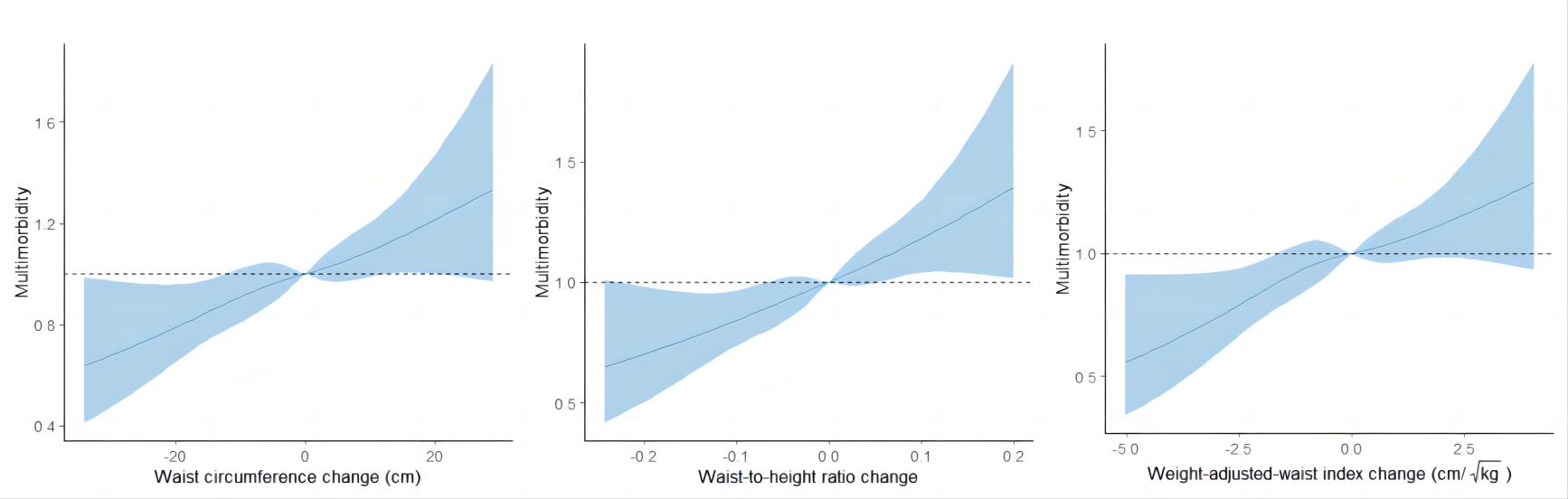


Supplementary Figure 6. Cubic splines of three-year changes in WC, WHtR, and WWI associated with multimorbidity (excluding participants with ADL disability).

Restricted-cubic-spline regression analysis with 4 knots was used to describe non-linear association of changes in waist circumference, waist-to-height ratio, and weight-adjusted-waist index with multimorbidity. Cox proportional hazards models were adjusted for age, sex, marital status, education level, residence, living pattern, occupation, household income, smoke, drink, exercise, sleep duration.


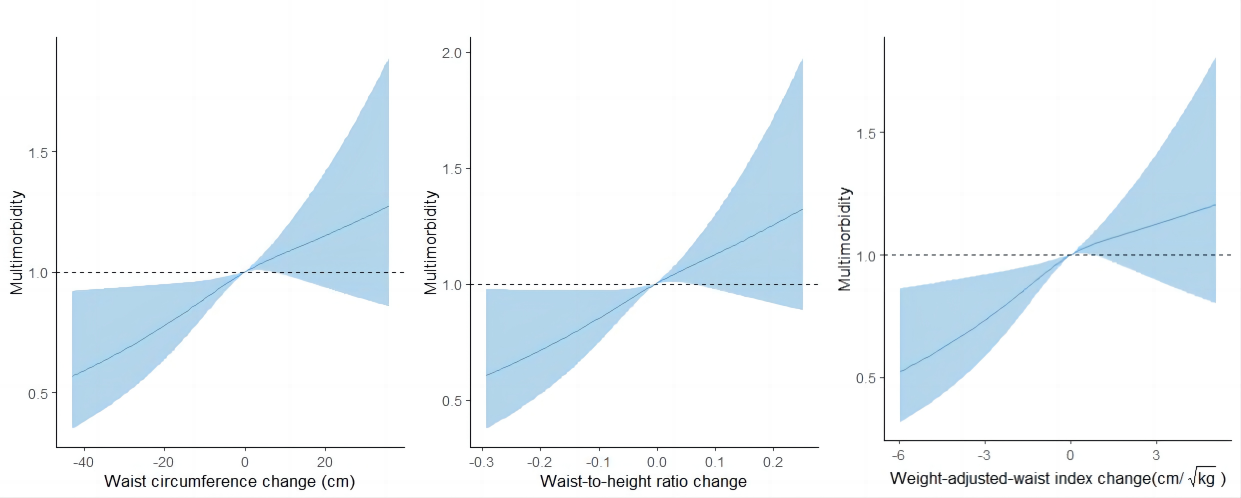


Supplementary Figure 7. Cubic splines of three-year changes in WC, WHtR, and WWI associated with multimorbidity (excluding participants with hypertension).

Restricted-cubic-spline regression analysis with 4 knots was used to describe non-linear association of changes in waist circumference, waist-to-height ratio, and weight-adjusted-waist index with multimorbidity. Cox proportional hazards models were adjusted for age, sex, marital status, education level, residence, living pattern, occupation, household income, smoke, drink, exercise, sleep duration.


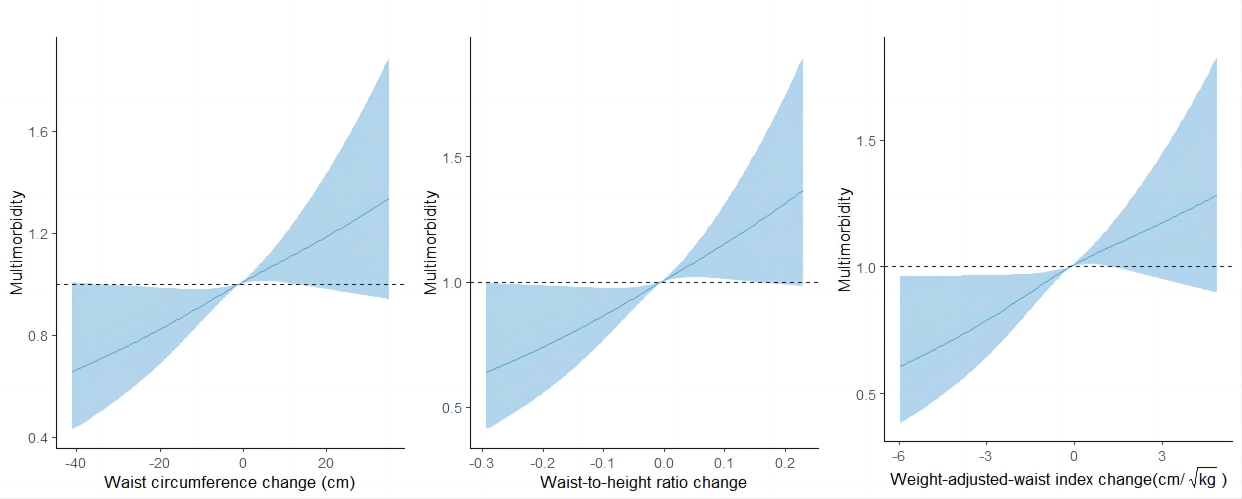


Supplementary Figure 8. Cubic splines of three-year changes in WC, WHtR, and WWI associated with multimorbidity (excluding participants with less than 2 years of follow-up).

Restricted-cubic-spline regression analysis with 4 knots was used to describe non-linear association of changes in waist circumference, waist-to-height ratio, and weight-adjusted-waist index with multimorbidity. Cox proportional hazards models were adjusted for age, sex, marital status, education level, residence, living pattern, occupation, household income, smoke, drink, exercise, sleep duration.
